# A Study for Development Suitability of Biomass Power Generation Technology Based on GHG Emission Reduction Benefits and Growth Potential

**DOI:** 10.1155/2022/7961573

**Published:** 2022-07-06

**Authors:** Deming Li

**Affiliations:** School of Economics and Management, Beijing Information Science and Technology University, Beijing, China

## Abstract

Biomass energy can alleviate global warming and solve energy depletion, which is increasingly concerned by the world. Due to the different emission reduction benefits and growth potential of different regions and biomass power generation technologies, analyzing the suitability of these technologies combined with regional conditions can more accurately guide the long- and short-term development of biomass power industry. However, there is no comprehensive evaluation of the benefits and potential of several biomass power generation technologies in different regions. Therefore, this paper introduces development suitability indexes and constructs life-cycle environmental impact and time-value economic impact assessment models, and a growth potential dynamic assessment model based on Gaussian process and particle swarm optimization to illustrate the greenhouse gas emission reduction benefits and growth potential of biomass power generation technologies. The empirical research of China shows that biomass gasification and direct combustion power generation can bring the best environmental benefits, and biogas power generation can bring the best economic benefits. For regions with abundant biomass resources and bad air condition, it is more suitable to develop gasification and direct combustion power generation technology, while mixed-combustion and biogas power generation technology are more suitable in regions with high electricity consumption.

## 1. Introduction

With the rapid development of economy and the continuous growth of population, the demand for electric energy is increasing. Fossil energy dominated by coal, natural gas, and oil has always been the main energy source in all countries. However, the depletion of fossil fuels and environmental deterioration has become the shackles of social development [1]. The transition to green and clean renewable energy is essential for the sustainable development of society. According to the latest BP world energy statistics yearbook (2021), the global primary energy consumption and the carbon emissions from consumption in 2020 are both recorded the largest decline since 1945, with primary energy consumption falling by 4.5%, oil consumption falling by nearly 3/4 of the net reduction, and carbon emissions falling by more than 6%. The installed capacity of wind and solar energy increases rapidly, with electricity generation increasing by 238,000 MW, 50% greater than any previous increase. The share of renewable energy in electricity generation increases from 10.3% to 11.7%, while the share of coal decreases by 1.3%. Renewable energy is maintaining strong growth.

Biomass has been a major source of energy since primitive times. Even in modern times, biomass has been the main fuel source for many developing countries [2]. As a kind of renewable energy, biomass has the characteristics of wide sources, abundant reserves, low emissions, and high application potential [3, 4], and is known as the fourth abundant energy after coal, natural gas, and oil [5]. Different from wind and solar energy, biomass stores solar energy in the form of chemical energy, and it uses agricultural residues as representative resources with low sulfur content, which can achieve carbon neutralization and effective emission reduction of GHG. Among the various energy conversion technologies, biomass power generation technology is relatively mature, and biomass power is of great quality and high reliability, which can generate electricity continuously without time limit, effectively avoiding the intermittent problems caused by wind and solar power generation [6, 7]. If biomass power generation technology can be developed, it can not only alleviate the current contradiction between energy shortage, environmental pollution and economic development, but also create income for farmers, such as creating jobs and increasing income, which is conducive to sustainable social development [8].

Biomass energy has been applied in almost every department of modern industry. The development policies and R&D priorities for biomass energy vary from country to country due to differences in resource conditions and environmental requirements [9]. In one country, the development strategies of the biomass power industry in different regions should also be suitably adjusted due to the differences in the technical level, environmental quality, resource conditions, and industrial demand. The long-term development of the biomass power industry requires a stable supply of resources, mature and viable technologies, and outstanding environmental and economic benefits [10]. Therefore, it is of great significance to analyze the economy and growth potential of biomass power generation technology combined with regional conditions, which can help different countries and regions develop biomass power industry by using their own advantages and achieve the goal of zero carbon emission to the greatest extent.

Research on biomass power generation is extensive, mainly focusing on resource evaluation, modeling, and optimization of power generation technology or process, and has made progress. For example, Song et al. [10] analyzed the greenhouse gas (GHG) emission reduction benefits of straw direct combustion power generation projects using cost-effectiveness analysis and market value method. Xu et al. [11] evaluated the environmental impacts of five mature biomass power generation technologies in China from the perspective of life cycle. Irfan et al. [12] assessed the power generation potential of biomass resources such as sugarcane residues, straw, and animal manure in Pakistan and predicted the development trend of biomass resources. Although these studies can provide suggestions for the overall development of biomass industry, they cannot be applied to a specific region. The guidance provided has some deviation due to the differences between regions. There is no systematic and comprehensive evaluation of the benefits and potential of several specific biomass power generation technologies based on different regions now. There are many kinds of biomass power generation technologies, and the economic benefits, environmental benefits, and development potential of GHG emission reduction of different power generation technologies are different. Specific analysis of the above indexes of different regions and power generation technologies can more accurately guide the long- and short-term development of biomass power industry.

Based on the above analysis, this paper uses the life-cycle assessment (LCA) method and dynamic analysis method based on time value [9] to illustrate the environmental and economic benefits of different biomass power technologies in different regions, and uses Gaussian process (GP) [13] and particle swarm optimization (PSO) algorithm [14] to predict and analyze the regional development potential of different biomass power technologies. The purpose of this paper is to provide reference for the biomass resources development strategies in different regions through benefit and growth potential evaluation.

Since China is a developing country with typical biomass power industry development characteristics, this paper focuses on empirical research of important biomass power generation technologies in China to analyze the GHG emission reduction benefits and growth potential of biomass power generation in four cities.

Compared with the existing work, the contributions of this paper can be summarized as follows.

1. It is the first time to analyze the GHG emission reduction benefits and growth potential of different biomass power generation technologies combined with the characteristics of one region.
2. Integrate the environmental and economic benefits of biomass power generation technology in the whole life cycle, and construct the benefit analysis model of GHG emission reduction.
3. A kernel function integrating long period kernel, short period kernel, rational kernel, and noise kernel is constructed, and GP is used to accurately fit the trend of environmental benefits of GHG emission reduction of biomass power generation technology.
4. The average annual growth rate of electricity consumption, the number of rural population, the noncompliance rate of air quality, and the amount of biomass resources in various regions are constructed as the development suitability indexes, and the growth potential of biomass power generation technology is dynamically evaluated by PSO.

The remaining sections of this paper are organized as follows.  Section 2 provides an overview and analysis of the existing research related to biomass resources.  Section 3 describes the data used in the empirical research in this paper.  Section 4 describes the economic and environmental benefits analysis methods, and the growth potential assessment methods of the GHG emission reduction of biomass power technology in detail.  Section 5 describes the empirical analysis process and results of biomass power generation technology in China.  Section 6 discusses the research results and gives some suggestions for the development of biomass.  Section 7 summarizes the work of this paper.

## 2. Related Work

In recent years, the research on biomass resources mainly focuses on the assessment of resource benefits and potential and GIS-based regional distribution analysis, the quantitative and sustainability evaluation of the environmental and economic impacts of biomass power generation technologies, and the macro policy suggestions on various factors affecting the development of biomass power industry [8].

### 2.1. Benefit and Potential Evaluation of Biomass Power Generation Resources

Biomass resources come from a wide range of sources and are mainly classified as forest residues, agricultural residues, animal and poultry manure, and domestic waste [8]. Different biomass resources are converted into electricity, heat, solid, and liquid fuels through various energy conversion technologies, and the GHG emissions in this process are different. The typical is the agricultural residue represented by straw, whose CO_2_ emissions during combustion are usually calculated as zero because they produce as much as CO_2_ absorbed during growth. In addition, there are differences in the processing costs and power generation potential of different biomass resources.

Many mature methods have been used to analyze biomass resources in terms of resource efficiency and potential assessment. Song et al. [10] calculated the incremental cost of GHG emission reduction of straw direct-fired power generation projects based on the international general calculation method of GHG emission reduction, and evaluated the benefits of straw direct combustion power generation projects in China using the cost-effectiveness analysis and the market value method. Irfan et al. [12] assessed the power generation potential of biomass resources such as sugarcane residues, straw, and animal manure in Pakistan and predicted the development trend of biomass resources. Xu et al. [15] developed a comprehensive model to analyze the logistics cost of straw recycling using calculus methods and guided the site selection planning of biomass power plants and straw planting mode in China through the cost value. Wang et al. [16] used ARIMA to predict the potential of agricultural biomass resources in Heilongjiang province and analyzed the trend of resource distribution on a spatial and temporal scale in combination with GIS. Ji et al. [17] predicted the agricultural residues in China based on ANN algorithm considering the change of crop sown area. Che [18] simulated the spatial distribution of straw resources in China using GIS technique and predicted the future resource potential using grey prediction method. The above research focus more on the energy utilization of regional biomass resources in the process of efficiency and potential calculation and spatial and temporal distribution analysis, ignoring the impact of the resource spatial distribution density on the energy production of biomass resources.

### 2.2. Evaluation of Biomass Power Generation Technology

The existing evaluation of biomass power generation technology mainly focuses on benefit and multi-index evaluation. Liang et al. [19] used cost-effectiveness analysis and market value approach to calculate the benefits of CO2 emission reduction, and compared with other renewable energy power generation technologies, concluded that attention should be paid to the development and research of biomass and other renewable energy power generation technologies. Xu et al. [11] evaluated the environmental impacts of five mature biomass power generation technologies in China from a whole life-cycle perspective. Malek et al. [20] summarized biomass scenarios in Southeast Asian and EU countries and used low, average, and high cost estimation methods to conduct economic analysis on biomass gasification power generation technology. Chen et al. [9] simulated and evaluated the environmental loads and economic benefits of biomass direct combustion, gasification, mixed-fired, and biogas power technologies in China using LCA and time-value-based dynamic analysis approach.

In addition, there are also a lot of researches that focus on the comparison of the development priorities of various biomass energy conversion technologies. For example, Zhou [21] used hierarchical analysis with ideal point method to integrate both biomass solid fuel and direct combustion power generation. Khishtandar et al. [22] combined the hesitant fuzzy language data and the preference of experts, adopted the multi-index method to deal with the priority of Iran's existing biomass technologies.

Most of the research on biomass power generation technologies focuses on modeling, optimization, and process evaluation of one technology or process in one region, and a few research focuses on analyzing the impact of specific multiple biomass power generation technologies on economic and environmental benefits. However, they did not explore the differences of regions, which are difficult to further guide the development of the biomass industry.

### 2.3. Macro Policy Analysis of Biomass Power Generation

The third perspective of biomass power generation is macro policy recommendation and strategy research, which qualitatively analyzes industrial development constraints and indirectly reflects the suitability of industrial development in different regions. The development of biomass power industry is influenced by various factors, such as cultivated land area, resource potential, logistics network, market demand, environmental demand, government support, business operation, and technology level [22–24]. Zhao et al. [25] used the five forces competition model to assess the current and future development of China's biomass power industry, and provided suggestions on procurement strategies for the sustainable development of the industry according to five influencing factors. Zhu et al. [26] used the strategic analysis tool in the SWOT-PEST model to explore the development of China's biomass power industry. Song et al. [27] constructed a biomass power potential index system to evaluate the development level and potential of biomass power generation in various regions in China, and optimized the target quotas for each city's planning installation by combining the carbon emission intensity of provincial power grids. The above research focuses on the exploration of some influencing factors in industrial development and fails to present the potential of biomass power industry development accurately and comprehensively.

It is found that the above research cannot systematically and comprehensively evaluate the GHG emission reduction benefits and growth potential of biomass power generation in different regions. They have one or more of the following problems.

1. There is no comprehensive assessment of multi biomass power technologies, but different power technologies have different benefits and development potential.
2. The analysis of biomass resources or power generation technology only for single region ignores the differences between regions, which is insufficient to guide the development of biomass power industry.
3. The assessment of the power generation potential of biomass resources or technologies takes into account historical data on single factor only, ignoring other influencing factors.

Based on the above analysis, we construct evaluation indexes for the suitability of biomass power technology development in terms of resource potential, development demand, and development conditions; conduct empirical research of the biomass power industry in four cities of China based on data from various yearbooks, literature, and some simulations to illustrate the environmental and economic benefits; and assess the growth potential of different biomass power technologies. The analytical approach adopted in this paper can be extended to different regions of other countries, better provide reference for the development strategy of biomass resources, and further promote the development of global biomass industry.

## 3. Data Description

The data used in this paper are obtained from China's statistical yearbook from 2012 to 2021 [28], the literature [9, 29–34], and some simulation data. The data include the life-cycle list of biomass power technologies, the capital data for biomass and coal-fired power plants, and data on development suitability indexes for four cities (A, B, C, D) in China. The specific data and related descriptions are given below.

### 3.1. Life-Cycle List of Power Generation Technologies

The life-cycle list contains the 1 kWh life-cycle GHG emissions of four major biomass power technologies (biomass direct combustion power generation, gasification power generation, mixed-fired power generation, and biogas power generation) and conventional coal-fired power technologies in China. This paper mainly considers the emissions of CO_2_ and CH_4_. The data are obtained from the literature [9, 29–34] and some simulation data. The emissions of CO_2_ and CH_4_ in 2020 of five power generation technologies in four cities are shown in Table 1.

### 3.2. Capital Inflow-Outflow List of Biomass Power and Coal-Fired Power Plants

We obtain various capital input-output data from the literature [9] and simulated data for representative biomass and coal-fired power plants in China and process them. The results are shown in Table 2.

The inflow capital during the construction and operation periods of the biomass power plant project we consider mainly includes by-product revenues such as electricity, and the outflow capital mainly includes civil construction, equipment procurement and installation, equipment commissioning and delivery, raw material and fuel procurement and transportation, labor management expenses, equipment depreciation, and other operating expenses. In data processing, the annual benchmark rate of return of the biomass power station is set to 10%, with an operating period of 20 years, an annual generating period of 5500 h, and a feed-in tariff of 0.8581 yuan/kWh, etc.

### 3.3. Development Suitability Index of Biomass Power Generation Technology

In this paper, we construct evaluation indexes for the suitability of biomass power technology development in terms of resource potential, development demand and development conditions, including the average annual growth rate of electricity consumption and the amount of biomass resources, the noncompliance rate of air quality, and the number of rural population. We select a representative city from North China, East China, Central China, and Southwest China, which are A, B, C, and D, respectively. Among them, B and C are large agricultural cities, A is an industrial city, and D is an animal husbandry city.

The electricity consumption value, cultivated land area, and forest area from 2010 to 2020 for four cities are obtained from China's statistical yearbook. The data from 2015 to 2020 are shown in Table 3. Among them, A and D have fewer rural laborers and smaller vegetation coverage and cultivated land area, which means that the biomass resources (such as straw) of the two cities are relatively less; B and C have more rural laborers and larger cultivated land area, especially C. Moreover, B and D have better air quality and lower carbon emissions; the power demand of D is growing faster than that of the other four cities. Due to the differences in development suitability index values, there are also some differences in the development of biomass technology between different regions. The purpose of this paper is to explore the most suitable biomass technology in one region based on the above indexes.

## 4. Methodology

In this paper, we construct a life-cycle environmental impact evaluation model and a time-value economic impact evaluation model to analyze the environmental and economic benefits of GHG emission reduction of biomass power generation technologies, and construct a growth potential dynamic assessment model to predict and explore the benefits and development potential of these technologies.

### 4.1. Life-Cycle Environmental Impact Assessment Model

Inspired by the literature [9], this paper uses LCA to analyze the whole life-cycle environmental assessment of biomass power generation and compare it with coal-fired power generation from conventional coal-fired power plants to describe the current status of environmental emissions and emission reduction benefits of various biomass power generation technologies and coal-fired power generation technology. Due to the complexity of systems involving multiple power generation technologies, the life cycle of these technologies considered in this paper includes only raw material production, processing, transportation, and power generation operation stages.

The calculation of GHG emission reduction benefits of different biomass power technologies in different regions through the LCA approach first requires the construction of inventory analysis data, i.e., gas pollutant emissions for each power technology over the full life-cycle process, which is interpreted by the intensity of the contribution of each specific environmental exchange to the determined environmental impact type. The data are described in detail in Section 3, as shown in Table 2.

Next, the potential environmental impact values are calculated for each impact type, assuming potential environmental impact value is *a* and GHG is *b*, the calculation equation is as follows:

(1) $$\begin{array}{c} EP\left(a\right)=\sum EP_{b}\left(a\right)=\sum \left[Q_{b}\times EF_{b}\left(a\right)\right], \end{array}$$

where *EP*(*a*) represents the contribution of *a*, *EP*_*b*_(*a*) represents the contribution of *b* to *a*, *Q*_*b*_ represents the emission of *b*, and *EF*_*b*_(*a*) is the equivalence coefficient of *b* and *a*.

With this equation, we calculate the total environmental load of different power generation technologies, and in this paper we only consider the environmental impact category of global warming.

### 4.2. Time-Value Economic Impact Evaluation Model

In this paper, we construct a model for calculating the economic benefits of biomass technologies based on the economic parameters of biomass power plant projects during the construction and operation periods. Inspired by the literature [10], we use dynamic analysis method based on time value to evaluate the economic benefits of different biomass power generation and coal-fired power generation technologies. The NPV will be used as a specific index to assess the economic benefits in this paper, which is calculated as follows:

(2) $$\begin{array}{c} \text{NPV}=\sum_{t=0}^{n}{\left(I-O\right)}_{t}{\left(1+i_{c}\right)}^{-t}, \end{array}$$

where *I* and *O* are capital inflow and capital outflow, respectively, (*I* − *O*)_*t*_ represents the net financial flows for the year *t*, *n* represents the total number of years, and *i*_*c*_ represents the base earning rate.

### 4.3. Model for Dynamic Assessment of Growth Potential

The existing research have not established systematic potential evaluation methods for different biomass power generation technologies, but generally the potential of one biomass power generation technology in one region and its application prospects are analyzed in terms of biomass resource, cost-effectiveness, economic conditions, and development demand, such as literature [35, 36].

The growth potential assessment of biomass power generation in this paper consists of two parts: one part is to predict the GHG emission reduction benefits (economic and environmental benefits) of important biomass power generation technologies in four regions of China based on GP, and the other part is to calculate the growth potential of different power generation technologies based on PSO.

The prediction of GHG emission reduction benefits of biomass power generation technologies is based on the emission reduction benefits of power generation technologies in the regions in the past years to predict the emission reduction benefits for the next years. GP is determined by mathematical expectations and covariance functions, and some covariance functions with specific forms become kernel functions. In this paper, we choose the sum of four kernel functions *K*=*K*_1_+*K*_2_+*K*_3_+*K*_4_ as the final kernel function of GP. *K*_1_, *K*_2_, *K*_3_, and *K*_4_ represent the long period kernel, short period kernel, rational kernel, and noise kernel functions, respectively. Since the GHG emission reduction benefits of biomass power generation technology may have short-term and long-term trends, *K*_1_ and *K*_2_ are used to fit this change, respectively; *K*_3_ is smooth, which can effectively fit the irregular parts; and *K*_4_ can model the residual noise in the data. Combining these four kernel functions, we can better capture the dynamic trend of emission reduction benefits. The equations of the four kernel functions are as follows:

(3) $$\begin{array}{c} K_{1}=\exp\left(-\frac{1}{\varepsilon_{1}^{2}}{\text{sin}}^{2}\frac{\pi \left(x_{i}-x_{j}\right)}{\lambda_{1}}\right),\\ K_{2}=\exp\left(-\frac{1}{\varepsilon_{2}^{2}}{\text{sin}}^{2}\frac{\pi \left(x_{i}-x_{j}\right)}{\lambda_{2}}\right),\\ K_{3}=\text{exp}{\left(1+\frac{{\left(x_{i}-x_{j}\right)}^{2}}{2\varepsilon_{3}^{2}}\right)}^{-\alpha },\\ K_{4}=\exp\left(-\frac{{\left(x_{i}-x_{j}\right)}^{2}}{2\varepsilon_{4}^{2}}\right), \end{array}$$

where *x*_*i*_ and *x*_*j*_ represent the emission reduction benefits in year *i* and *j*, respectively; *ε* are developed to control how fast the kernel function changes with time; and *λ* is the cycle length.

After the kernel function configuration is completed, this paper solves the mathematical expectation, and kernel function of the Gaussian joint distribution is resolved by maximizing the log-likelihood function of the hyperparameters. Assuming that *m*(*t*) is the mathematical expectation and *K*(*t*) is the covariance, the equation is as follows:

(4) $$\begin{array}{c} \text{log}P\left(x_{t+1}|X,m\left(t\right),K\left(t\right)\right)=-\frac{1}{2}\text{log}\left|K\left(t\right)\right|-\frac{1}{2}{\left(x_{t+1}-m\left(t\right)\right)}^{T}K{\left(t\right)}^{-1}\left(x_{t+1}-m\left(t\right)\right). \end{array}$$

**Figure 1:**
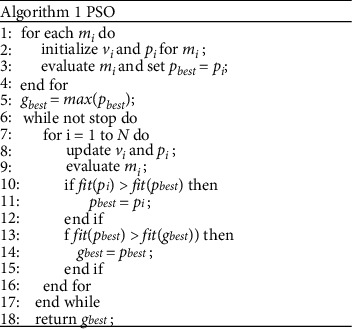
Flow of PSO.

In order to quantify the long-term joint impact of different indexes on the growth potential of biomass power generation technology, this paper uses PSO to achieve the goal [14]. PSO simulates the birds in the bird swarm through a massless particle. The current position of the particle is a candidate solution to the corresponding optimization problem, the flight process is the search process, and the flight speed can be dynamically adjusted according to the historical optimal position of the particle and the swarm. Each particle has two attributes of position and velocity. The optimal solution searched separately is called individual extremum, and the optimal individual extremum in the particle swarm is used as the current global optimal solution. In the process of iteration, the attributes of each particle are updated, and finally the optimal solution satisfying the termination condition is obtained. The flow of the algorithm is shown in Figure 1. Here, *m*_*i*_ represents a random particle, *v*_*i*_ and *p*_*i*_ are the velocity and position attributes of the particle, respectively, and *p*_best_ represents the combination of historical optimal development suitability indexes for a single particle, and *g*_best_ represents the combination of historical optimal development suitability indexes for a particle swarm. In this paper, the algorithm is used to mine the maximum GHG emission reduction benefits of biomass power technology and obtain the best combination of development suitability indexes, so the search objective of the particle swarm in this paper is to solve the maximum benefit. The algorithm first initializes *N* random particles *m*_*i*_ in the feature space. In the iteration *S*, *m*_*i*_(*s*)=(*m*_*i*,1_(*s*),…, *m*_*i*,*d*_(*s*),…, *m*_*i*,*D*_(*s*), where *D* represents the number of development suitability indexes used in this paper. We use CfsSubsetEval to evaluate the current combination of developmental suitability indexes of the particles, and the output value F(*m*_*i*_(*s*)) is used as the evaluation index of *m*_*i*_(*s*). Since the objective is to solve the benefit maximization problem, the larger the F(*m*_*i*_(*s*))) of *m*_*i*_(*s*), the better the position it is than other particles. During the iteration, the particles always maintain *p*_best_ and *g*_best_.

In this paper, PSO is used to find the best indexes combination that makes the biomass power generation technologies get the greatest environmental benefits. Then, we can get the factors that affect the development of one technology, and the growth potential in one region can be accessed based on the regional characteristics.

## 5. Case Study and Result Analysis

Taking China as an example, this paper analyzes the benefits of biomass direct combustion, gasification, mixed-fired and biogas power generation technologies in four cities (A, B, C, and D) from both environmental and economic perspectives, and introduces development suitability indexes to predict development trends and assess growth potential of these technologies in these cities. To calculate the emission reduction benefits, we introduce coal-fired power generation technology.

### 5.1. Environmental Benefit Analysis of Biomass Power Generation Technology

We select unit capacities of 30 MW, 4 MW, 300 MW, 2 MW, and 1,320 MW for biomass direct combustion power generation, gasification power generation, mixed-combustion power generation, biogas power generation, and coal-fired power generation, respectively, set the lifetime of all power generation projects to 20 years, and set the functional unit (Fu) as power generation 1 kWh.

In this paper, global warming is selected as an environmental impact category and assess the impact of GHG emissions from each power generation technology on global warming because the measured impact potential of this impact category is close to the true potential and the assessment is more accurate and representative [9]. Global warming is calculated in terms of CO2-equivalents. The characterization factor of CO2 is 1 and that of CH4 is 25.

According to Table 1 and equation (1), in 2020, the potential GHG impact values for each power generation technology are calculated as shown in Table 4. Taking the potential impact value of direct combustion power generation technology of city A as an example, the calculation process of these results is: the emissions of CO_2_ and CH_4_ are obtained from Table 1, which are, respectively, 3.30 × 10^−3^ and 2.30 × 10^−6^, then the potential impact value is 3.30 × 10^−3^ × 1+2.30 × 10^−6^ × 25=3.35 × 10^−3^.

According to Table 4, in 2020, the potential impact of gasification power generation technology on GHG is the least in each city, followed by direct combustion, biogas, and mixed-combustion power generation. Compared to the environmental load of conventional coal-fired power generation, their average environmental emission reduction benefits are 95.52%, 93.29%, 54.73%, and 19.79%, respectively.

**Figure 2:**
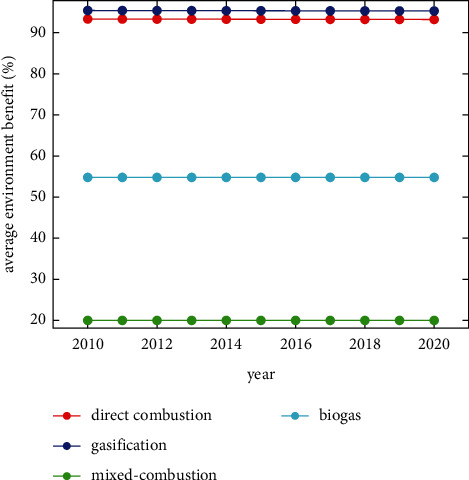
Average environmental benefits of four biomass technologies in China from 2010 to 2020.

In addition, this paper calculates the average environmental emission reduction benefits of each biomass power generation technology in A, B, C, and D (relative to coal-fired power generation technology) from 2010 to 2020, as shown in Figure 2. Overall, the environmental benefits of the four technologies maintain a relatively stable trend. The benefits of gasification and direct combustion power generation technologies are high, the benefits of biogas power generation are general, and the benefits of mixed-combustion power generation are relatively low.

### 5.2. Economic Benefit Analysis of Biomass Power Generation Technology

The capital input-output data we used for biomass and coal-fired power plants are shown in Table 2, and the contents of which are described in detail in Section 3.

The economic efficiency index considered in this paper is NPV. According to Table 2 and equation (2), the economic efficiency indexes for each generation technology are shown in Table 5.

Taking the economic benefit calculation process of direct combustion power generation technology of city *A* as an example: *I*=141,506,266+60,547,530 =202,053,796; *O*=306,453,975 + 80,958,737=441,412,712; then the value of NPV is ∑_*t*=0_^20^(202,053,796 − 441,412,712)_*t*_(1+0.1)^−*t*^= 6,963,824.

Based on the results of the economic evaluation of different biomass power technologies in different cities, on the whole, the technology for biomass biogas power has the highest NPV, followed by direct combustion power, coal-fired power technology, and lower NPV for mixed-combustion power and gasification power. In the four cities, direct combustion and biogas power generation have greater economic benefits compared to coal-fired power generation.

### 5.3. Development Trend Prediction of Biomass Power Generation Technology

In this paper, we analyze the development trend of different biomass power generation technologies in each city from an environmental perspective, and use the environmental benefits of GHG emission reduction of the important biomass power generation technologies in four cities of China A, B, C, and D from 2010 to 2020 to predict the environmental benefits of each technology in each city from 2021 to 2030. To ensure the accuracy of the results, we first divide the data from 2010 to 2020 into training set and test set for experiments to evaluate the prediction performance of GP. Among them, the data from 2010 to 2017 are used as the training set and the rest data are used as the test set. The experimental results are shown in Table 6. It can be seen that the average prediction accuracy of GP is 86%, which can effectively fit the trend of environmental benefits of various biomass power generation technologies.

**Figure 3:**
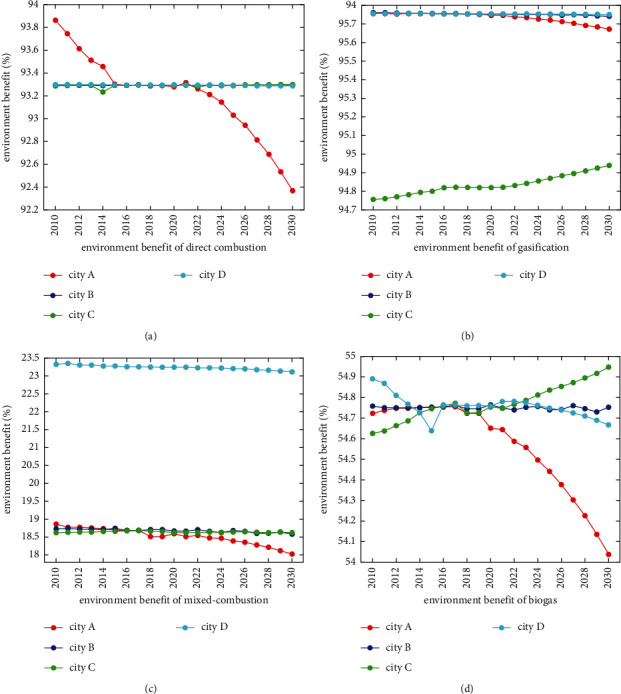
Environmental benefits of GHG emission reductions for four important biomass power technologies in four cities of China from 2021 to 2030. (a) Environment benefit of direct combustion. (b) Environment benefit of gasification. (c) Environment benefit of mixed-combustion. (d) Environment benefit of biogas.

Next, we use the trained GP model to predict the environmental benefits of four biomass power generation technologies in four cities from 2021 to 2030, as shown in Figure 3. The environmental benefits of these technologies vary greatly, especially biogas power generation technology. For direct combustion power generation, A shows an obvious downward trend, while B, C, and D remain unchanged; for gasification power generation, A decreases slightly and C increases significantly; the environmental benefits of hybrid and biogas power generation technology in A and D show a slight downward trend; C shows an upward trend of biogas power generation year by year, and B fluctuates slightly but remains basically unchanged. From 2021 to 2030, biomass power generation technology with the best environmental benefits in the four cities is still gasification and direct combustion, followed by biogas and hybrid power generation.

### 5.4. Assessment of Growth Potential of Biomass Power Generation Technologies

This paper introduces the average annual growth rate of electricity consumption and the amount of biomass resources biomass resources, the air quality noncompliance rate, and the number of rural population as indexes affecting the development of biomass power generation technology in one city, where the average annual growth rate of electricity consumption and biomass resources reflect the resource potential of a city, the air quality noncompliance rate reflects the need for environment improvement in the city, and the number of rural population reflects the agricultural biomass cultivation, collection, and human conditions in the transportation process.

We construct a PSO model to search for the best impact indexes of each biomass power technology based on the data of environmental benefits and development suitability indexes, so as to analyze the most suitable biomass power technology to be developed in each city. We construct the model on WEKA 3.9.5 and iterate 20 times to obtain the results as shown in Table 7.

According to Table 7, the air quality noncompliance rate and the amount of biomass resources are the main factors affecting the biomass direct combustion and gasification power generation technologies, and the annual growth rate of electricity consumption is the main factor affecting the mixed-combustion and biogas power generation technologies. Combining Tables 3 and 7, city A and city C have higher air quality noncompliance rates and city C has abundant biomass resources, so biomass direct combustion power generation and gasification power generation technologies have greater development potential in these two cities, which confirms our previous conclusion that these two technologies have the best environmental benefits in terms of GHG emission reduction. The average annual growth rate of electricity consumption in city B and D is much higher than that of other cities, which indicates that the demand for electricity in these two cities is high, so the mixed-combustion and biogas power generation technologies have greater development potential in city B and C. In addition, city B also has abundant biomass resources, so it is also suitable for developing biomass direct combustion and gasification power generation technologies.

## 6. Discussion and Policy Recommendations

Biomass power generation has become a great need for sustainable development and clean energy. However, due to the differences of technical level and environmental quality in various regions, the biomass power generation technology suitable for development is also different. Only by analyzing the economic benefits, environmental benefits and growth potential of biomass power generation technology in combination with regional conditions, can we promote the development of biomass industry in various regions and achieve the goal of zero emission to the greatest extent. For China dominated by agriculture, biomass gasification power generation and direct combustion power generation technology can bring better environmental benefits, and biogas power generation technology can bring better economic benefits. Specifically, cities with serious air pollution in China, development priority should be given to biomass power generation technologies, such as direct combustion power generation and gasification power generation, if there are a large number of local rural areas and abundant biomass resources such as straw and forestry waste, it is also more suitable for the development of these two biomass industries. For areas with high electricity consumption, development priority should be given to the promotion of mixed-combustion and biogas power generation technology.

Based on our research findings, the following policy recommendations are given for the China government.

1. Establish suitability indicators for the development of biomass industry: the government should divide regions according to the average annual growth rate of electricity consumption, biomass resources, air quality noncompliance rate, rural population and other indicators, and make development strategies according to local conditions.
2. Further stimulate people's environmental awareness: the government should educate the public about the benefits of biomass for sustainable development, especially for rural residents, as they are located in regions with sufficient biomass resources, they should be educated to make the best of straw, livestock manure, and other resources to avoid resource waste.
3. Explore new markets and technologies: the government should promote the export of technologies and products of local biomass energy enterprises. For example, for agricultural countries such as Southeast Asia, direct combustion power generation and gasification power generation technology can be promoted. In addition, the government should constantly absorb modern technology and enhance the economic and environmental benefits of biomass technology.

## 7. Conclusion

In this paper, the environmental load and economic benefits of the biomass power generation process are simulated using the LCA and the dynamic analysis method based on time value, and the environmental and economic benefits of different biomass power generation technologies are illustrated by comparing them with coal-fired power generation technologies. In addition, the GP and PSO algorithm are used to predict and analyze the development potential of different biomass power generation technologies in different regions. According to the empirical analysis of China, biomass direct combustion and gasification power generation technology are suitable for development in regions with concentrated biomass resources and serious air pollution; mixed-combustion and biogas power generation technology are suitable for use in regions with high electricity demand. China's geographical situation and resource characteristics determine that it should vigorously develop biomass gasification and direct combustion power generation technology, and develop mixed-combustion and biogas power generation technology according to local conditions. Although the research of this paper focuses on the agricultural countries represented by China, it is also beneficial to other typical geographical patterns and can effectively guide the development of the biomass industry in different regions of other countries.

## Figures and Tables

**Table 1:** Life-cycle list of different generation technologies.

City	Technology	CO_2_(kg/kWh)	CH_4_(kg/kWh)

A	Direct combustion	3.30 × 10^−3^	2.30 × 10^−6^
	Gasification	2.10 × 10^−3^	2.10 × 10^−6^
	Mixed-combustion	3.80 × 10^−2^	9.00 × 10^−5^
	Biogas	2.20 × 10^−2^	2.40 × 10^−6^
	Coal-fired	4.70 × 10^−2^	1.10 × 10^−4^

B	Direct combustion	2.21 × 10^−2^	1.54 × 10^−5^
	Gasification	1.39 × 10^−2^	1.39 × 10^−5^
	Mixed-combustion	2.57 × 10^−1^	6.20 × 10^−4^
	Biogas	1.51 × 10^−1^	1.60 × 10^−5^
	Coal-fired	3.16 × 10^−1^	7.70 × 10^−4^

C	Direct combustion	1.24 × 10^−2^	8.60 × 10^−6^
	Gasification	7.70 × 10^−2^	7.80 × 10^−5^
	Mixed-combustion	1.44 × 10^−1^	3.50 × 10^−4^
	Biogas	8.40 × 10^−2^	8.90 × 10^−6^
	Coal-fired	1.77 × 10^−1^	4.30 × 10^−4^

D	Direct combustion	6.11 × 10^−2^	4.26 × 10^−5^
	Gasification	3.88 × 10^−2^	3.87 × 10^−5^
	Mixed-combustion	6.77 × 10^−1^	1.62 × 10^−5^
	Biogas	4.17 × 10^−1^	4.41 × 10^−5^
	Coal-fired	8.70 × 10^−1^	2.12 × 10^−3^

**Table 2:** Capital inflow-outflow list of different power generation technologies.

City	Technology	Initial investment (yuan)	Operation investment (yuan)	Operation profit (yuan)	Annual profit (yuan)	Initial investment (yuan)

A	Direct combustion	306,453,975	80,958,737	141,506,266	60,547,530	306,453,975
	Gasification	66,162,041	10,988,891	18,867,502	7,878,612	66,162,041
	Mixed-combustion	1,370,752,620	1,011,435,335	1,061,451,368	50,016,033	1,370,752,620
	Biogas	110,151,908	3,716,326	19,862,334	16,146,008	110,151,908
	Coal-fired	6,031,345,832	3,081,074,539	3,882,428,813	801,354,274	6,031,345,832

B	Direct combustion	245,163,180	64,766,989	113,205,013	48,438,024	245,163,180
	Gasification	52,710,791	8,791,111	15,094,002	6,302,891	52,710,791
	Mixed-combustion	109,660,173	809,148,264	849,161,092	40,012,828	109,660,173
	Biogas	88,121,530	2,973,061	15,889,864	12,916,803	88,121,530
	Coal-fired	4,825,076,669	2,464,859,630	3,105,943,049	641,083,419	4,825,076,669

C	Direct combustion	220,646,862	58,290,287	101,884,512	43,594,198	220,646,862
	Gasification	47,439,708	7,912,004	13,584,602	5,672,606	47,439,708
	Mixed-combustion	98,694,152	728,233,434	764,244,986	36,011,543	98,694,152
	Biogas	79,309,373	2,675,755	14,300,875	11,625,121	79,309,373
	Coal-fired	79,309,373	2,218,373,664	2,795,348,747	576,975,074	79,309,373

D	Direct combustion	147,097,908	38,860,197	67,923,008	29,062,811	147,097,908
	Gasification	31,626,478	5,274,666	9,056,401	3,781,735	31,626,478
	Mixed-combustion	65,796,098	485,488,962	509,496,657	24,007,695	65,796,098
	Biogas	52,872,922	1,783,837	9,533,920	7,750,083	52,872,922
	Coal-fired	2,895,046,001	1,478,915,776	1,863,565,831	384,650,055	2,895,046,001

**Table 3:** Biomass power generation technology development suitability indexes for four cities in China from 2015 to 2020.

Year	City	Average annual growth rate of electricity consumption (%)	Number of rural population	Air quality noncompliance rate (below secondary) (%)	Amount of biomass resources

2015	A	1.67	293	49.04	218.7
	B	6.75	2209	14.79	1618.2
	C	−1.36	5039	62.74	4756.9
	D	19.28	234	14.25	70.1

2016	A	1.67	293	49.18	211.8
	B	6.75	2,209	15.03	1,620.6
	C	−1.37	5,039	62.84	4,745
	D	10.93	234	14.48	70.1

2017	A	7.09	293	45.75	197.4
	B	8.76	2,154	12.88	1,771.5
	C	3.80	4,909	56.44	4,698.5
	D	21.44	233	14.25	54.6

2018	A	4.60	293	38.08	188.6
	B	9.38	2,098	17.81	1,785.8
	C	5.92	4,764	54.52	4,681.6
	D	18.37	233	1.10	81.3

2019	A	7.03	291	37.81	209.8
	B	10.43	2,044	10.41	1,868.8
	C	7.96	4,638	53.97	5,102.7
	D	18.97	237	1.92	91.3

2020	A	2.10	289	34.25	217.9
	B	7.49	1,987	11.78	1,967.1
	C	−1.58	4,511	51.51	5,549.4
	D	13.04	240	6.30	98.4

**Table 4:** Characterization results of different power generation technology in 2020.

Technology	City A	City B	City C	City D

Direct combustion	3.35 × 10^−3^	2.25 × 10^−2^	1.26 × 10^−2^	6.22 × 10^−2^
Gasification	2.13 × 10^−3^	1.42 × 10^−2^	9.74 × 10^−3^	3.94 × 10^−2^
Mixed-combustion	4.07 × 10^−2^	2.73 × 10^−1^	1.53 × 10^−1^	7.11 × 10^−1^
Biogas	2.27 × 10^−2^	1.52 × 10^−1^	8.51 × 10^−2^	4.19 × 10^−1^
Coal-fired	4.50 × 10^−2^	3.35 × 10^−1^	1.88 × 10^−1^	9.27 × 10^−1^

**Table 5:** Economic evaluation results of different power generation technologies.

City	Technology	Scale (MW)	NPV (yuan·MW^−1^)

A	Direct combustion	30	6,963,824
	Gasification	4	228,697
	Mixed-combustion	300	1,417,921
	Biogas	2	13,653,211
	Coal-fired	1,320	5,168,552

B	Direct combustion	30	5,571,061
	Gasification	4	182,958
	Mixed-combustion	300	1,134,339
	Biogas	2	10,922,570
	Coal-fired	1,320	4,134,838

C	Direct combustion	30	5,013,954
	Gasification	4	164,665
	Mixed-combustion	300	1,020,902
	Biogas	2	9,830,314
	Coal-fired	1,320	3,721,360

D	Direct combustion	30	3,342,633
	Gasification	4	109,771
	Mixed-combustion	300	680,602
	Biogas	2	6,553,537
	Coal-fired	1,320	2,480,906

**Table 6:** Environmental benefits prediction accuracy of GP.

\	Train	Test

Accuracy (%)	87.89	84.73

**Table 7:** Economic evaluation results of different power generation technologies.

Technology	Indexes

Direct combustion	Air quality noncompliance rate, amount of biomass resources
Gasification	Air quality noncompliance rate, amount of biomass resources
Mixed-combustion	Annual growth rate of electricity consumption
Biogas	Annual growth rate of electricity consumption

## Data Availability

All data could be accessed by request.
